# Comparative Analysis of the Phase Interaction in Plasma Surfaced NiBSi Overlays with IVB and VIB Transition Metal Carbides

**DOI:** 10.3390/ma14216617

**Published:** 2021-11-03

**Authors:** Mariusz Bober, Jacek Senkara, Hong Li

**Affiliations:** 1 Faculty of Mechanical and Industrial Engineering, Warsaw University of Technology, Narbutta 85, 02-524 Warsaw, Poland; jacek.senkara@pw.edu.pl; 2 Faculty of Materials and Manufacturing, Beijing University of Technology, Beijing 100124, China

**Keywords:** transition metal carbides, NiBSi alloy, phase interaction, PTAW, composite layers

## Abstract

Important applications of transition metal carbides (TMCs) are as wear resistant composite layers deposited by plasma transferred arc welding (PTAW) and laser methods. Growing interest in them has also been observed in additive manufacturing and in HEA technology (bulk composite materials and layers), and in the area of energy conversion and storage. This paper presents the results of comparative studies on interfacial interactions in the NiBSi−TMCs system for two border IVB and VIB TM groups of the periodic table. Model (wettability and spreadability) and application experiments (testing of the PTAW-obtained carbide particle−matrix boundaries) were performed. Fe from partially melted steel substrates is active in the liquid NiBSi−TMCs system. It was revealed that the interaction of TMCs with the liquid NiBSi matrix tends to increase with the group number, and from the top to bottom inside individual groups. Particles of IVB TMCs are decomposed by penetration of the liquid along the grain boundaries, whereas those of VIB are decomposed by solubility in the matrix and secondary crystallization. No transition zones formed at the interfacial boundaries of the matrix−IVB group TMCs, unlike in the case of the VIB group. The experimental results are discussed using the data on the TMC electronic structure and the physicochemical properties.

## 1. Introduction

Surface composite layers (SCLs) consisting of a metal matrix strengthened with hard particles of high-melting phases (simple carbides, borides, nitrides, or more complex phases) combine the properties of an abrasion-resistant and relatively plastic matrix and hard ceramics, often with the effect of synergy. Such coatings can be deposited onto large surfaces, on selective areas (flat or curved), or on the edges. They meet the industrial needs wherever high wear resistance is required at parallel dynamic loads, often in corrosive environments and in elevated temperatures. They have been known and used for a relatively long time. Despite the fact that almost all welding methods can be applied to obtain such SCLs, they are usually obtained from powders by padding, re-melting, or thermal spraying techniques. Today, the implementation of laser and plasma beams is mainly used for this purpose (see [1,2,3,4,5], for instance). In general, variants of all of the following methods are utilized in the production of SCLs [6,7,8,9,10]:

- injection of refractory particles to the previously initiated liquid weld pool,
- simultaneous introduction of metal−ceramic powder mixtures to the beam or arc,
- melting pre-placed powder mixtures at the surface,
- strengthening of the matrix by in-situ formed particles.

During the preparation of SCLs, regardless of the method, an interaction occurs between the solid refractory particles and the liquid matrix material. As a result, interfacial boundaries are formed. This is the case for all types of upper layer formations in the presence of a liquid phase, namely alloying, buttering, cladding, overlaying, and hardfacing. Phenomena at the interfaces are crucial from the point of view of layer formation and its subsequent exploitation [11,12,13]. If the strengthening phase particles are wetted well with the liquid matrix, a good adhesion and low interfacial energy are achieved, and they are kept in the liquid pool. Their distribution in the matrix also depends on wettability. The particle−matrix interface type depends on the chemical affinity of the contacting phases and can be formed as a result of an adhesive (in inert systems) or more complex interaction in the diffusion or in the reaction-controlled systems [14]. The type of resulting interface—with or without an intermediate zone—is important for the sake of the load transfer to the strengthening phase and the level of residual stresses, and hence for the behavior of the whole SCL under the external load. Moreover, in the case of service at elevated temperatures, the transition zone may enlarge, change, or even degrade due to the volume or reactive diffusion.

Recently, there has been increased interest in SCLs with transition metal carbides (TMCs). An impetus was brought about by the new applications in other advanced technologies. Apart from being used as only surface layers of machine parts, they have found applications in additive manufacturing technologies for the production of massive 3D printed wear resistance parts using laser or electron beams [15,16]. Their new capabilities and increasing interest are also related to the applications of TMCs in the areas of energy storage and conversion in batteries and fuel cells [17,18], as well as in catalysis [19,20]. Another developing direction is a new class of high-entropy materials of composite structure containing TMCs [21,22,23,24,25]. This causes a need for more in-depth research on composites with TMCs. While many publications in the field have focused on the methods of producing SCLs, their structure and tribological properties, computer modeling, and process simulation, much less information has emerged about the interfacial interactions.

The aim of this work is to analyze the interaction between the matrix and individual carbides in the plasma transferred arc welding (PTAW) process so as to find the regularities resulting from the position of the transition metal forming the carbide in the periodic table of elements. This paper presents the results of comparative studies on interfacial interactions in the system of NiBSi-TMCs for two border TM groups (IVB and VIB). Basic and application experiments were performed for this purpose. The first relied on the investigation of the wetting and spreading of the liquid alloy droplets on a flat carbide surface at a macroscopic scale under controlled temperature conditions. In the framework of application research, composite overlays were made from powders using the PTAW method with subsequent testing of the carbide particles–matrix boundaries. During the technological process, a dynamic interaction occurred between the liquid phase and the curved surfaces of the small strengthening particles.

The NiBSi alloy was selected as the matrix material for the SCLs because it is produced commercially in the form of a powder for the manufacture of overlays that exhibit excellent corrosion, abrasion, and wear-resistance at up to 600 °C [26]. The alloy comes from the Ni-rich corner of the Ni-B-Si system, but with a relatively low melting range due to the presence of B and Si additives that act as a melting point depressants [27].

## 2. Materials and Methods

### 2.1. Materials

Two forms of materials were applied in the research:

- Specially prepared massive solids for the model wettability and spreadability tests;
- Commercial powders for plasma surfacing.

#### 2.1.1. Composite Matrix Material

**Figure 1:**
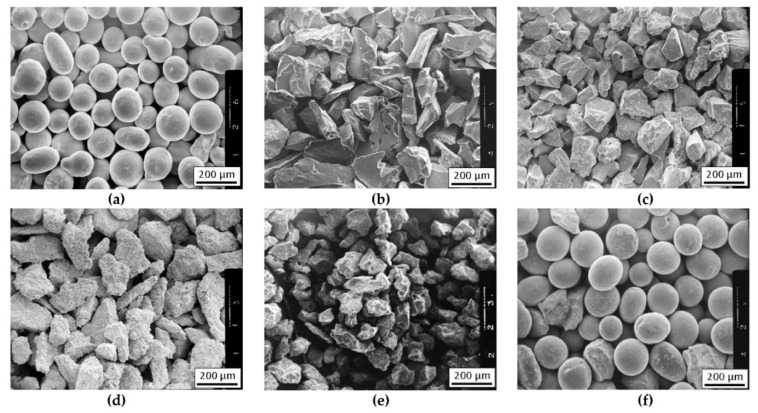
The morphology of powders for the production of composite layers using the plasma surfacing method: (**a**) NiBSi, (**b**) TiC, (**c**) ZrC, (**d**) Cr_3_C_2_, (**e**) Mo_2_C, and (**f**) WC.

The nominal chemical composition of the NiBSi alloy of the purchased powder according to the manufacturer certificate is presented in Table 1, along with the composition determined by the atomic absorption spectrophotometry (AAS) method. The granularity of this powder was in the range of 45 ÷ 150 μm. Its morphology is presented in Figure 1a.

The solid alloy of the corresponding composition was used in the wettability and spreadability tests. It was obtained by melting the pure elements in a vacuum induction furnace. In order to avoid segregation of the components during the ingot crystallization and following troublesome plastic forming, the liquid melt was sucked off by pure Ar gas directly from the crucible into quartz tubes with an internal diameter of 3 mm. The rods formed in this way were cut into pieces with equal lengths of 3 mm. The compatibility of the chemical composition of the material produced in this way with the commercial powder was confirmed by the AAS analysis (Table 1). The solidus and liquidus temperatures of the obtained alloy, determined by the differential thermal analysis (DTA) method, were 1075.5 and 1086 °C, respectively.

#### 2.1.2. Strengthening Carbide Phase

The TMCs powders of the IVB and VIB group of the periodic table were used for the strengthening phase. Commercial powders of carbides of titanium (TiC), zirconium (ZrC), chromium (Cr_3_C_2_), molybdenum (Mo_2_C), and tungsten (WC), of a similar grain size 80–200 μm, were used for the preparation of the composite layers. The morphology of the particular powders is shown in Figure 1b–f. Carbide powders were mixed with the matrix NiBSi powder before hardfacing.

**Figure 2:**
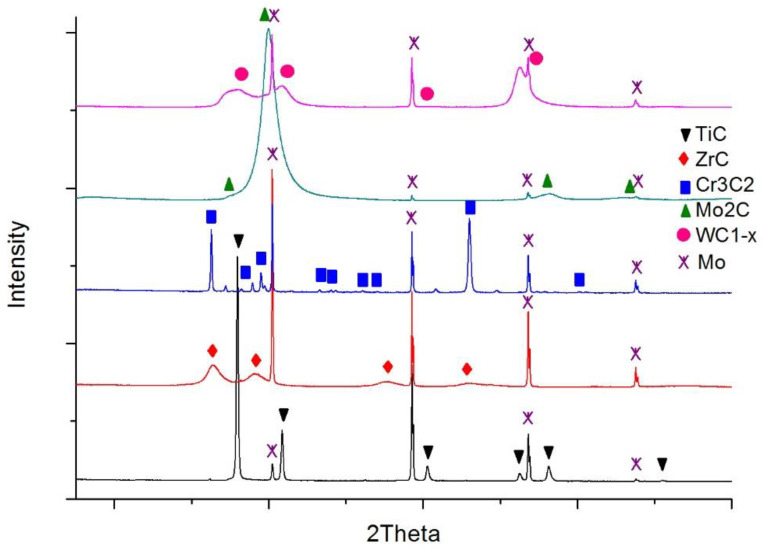
XRD patterns of the carbide layers over molybdenum surfaces.

The carbide substrates for the wetting and spreading tests were made in the form of continuous layers over molybdenum plates using the magnetron sputtering method. In this way, possible errors due to the harmful impact of activators and residual oxide inclusions on wettability were avoided, if commercial sintered carbide materials were used instead. Mo plates were selected due to their high melting point (2883 K) and low thermal expansion coefficient (λ = 4.9 × 10^−6^ K^−1^) [28] so as to prevent a high stress level at the boundary with the carbide layers. Then, 18 × 18 mm plates were cut from a 1 mm thick molybdenum sheet and polished. These prepared substrates were covered with coatings of five different carbides (i.e., titanium, zirconium, chromium, molybdenum, and tungsten carbide) in the process of magnetron sputtering of suitable metal targets in the reactive gas atmosphere (mixture of argon and acetylene). The details of the whole procedure are described in [29]. As a result, continuous coatings adhesively bonded to the molybdenum plates were obtained. The thicknesses of these coatings are presented in Table 2. Measured surface roughness was small and comparable reflecting the roughness of the Mo plates (R_a_ parameter < 0.8 μm for all cases). X-ray diffraction (XRD) phase analysis confirmed the composition of the five planned carbides (Figure 2).

#### 2.1.3. Substrate Material for Plasma Surfacing

The base material applied was 1.0553 grade (S355J0) low-alloy steel in the form of 10 × 50 × 150 mm plates.

### 2.2. Methods

#### 2.2.1. Wettability and Spreadability Test

The study was carried out by the sessile drop method in strictly controlled conditions. The substrate samples (18 × 18 × 1 mm Mo plates with deposited carbide layers) and NiBSi alloy (ϕ3 × 3 mm cylinders) were washed in an ultrasonic cleaner in acetone and were placed on a leveled measuring table in the chamber of the heating device. Due to the fact that plasma surfacing process was performed in an argon shield, the wetting and spreading tests were also carried out in an argon atmosphere with a high 99.999% purity. The working chamber of the device was pumped down to a pressure of 10 Pa, and then filled with argon. This treatment was repeated twice to thoroughly remove any residual air. Then, a constant argon flow of 0.5 L/min was established. The samples were heated from an ambient temperature to 900 °C at a rate of 100 °C/min, and then the rate was reduced to 20 °C/min. The measurement consisted of registering the shape of the drop contour from the time of melting every 10 °C as the temperature increased. The contact angles were measured on both sides of the droplets and the results were averaged. The processes were terminated after the temperature reached 1350 °C. The samples were cooled then and taken off the device. The areas of drop spread were measured. Two such tests were carried out for each NiBSi alloy–carbide substrate system.

#### 2.2.2. Plasma Surfacing

Padding weld samples were made using the plasma transferring arc welding (PTAW) method. The NiBSi matrix powder was mixed with each carbide powder in a volume ratio of 60:40 prior to the process. Before starting, each of the substrate samples (1.0553 grade steel) was sandblasted and cleaned with acetone. The PTAW process was carried out at different current values of the main arc whereas all remaining parameters were constant (Table 3). There was no pre-heating. All of the overwelds had the same length of 60 mm.

The structural tests were carried out on specimens in the plane perpendicular to the weld axis. The samples were cut by means of an electrical discharge cutter at the same distance of 20 mm from the beginning of each weld.

## 3. Results and Discussion

### 3.1. Interaction in the Model System of Liquid NiBSi Alloy-Solid Carbide Surface

**Figure 3:**
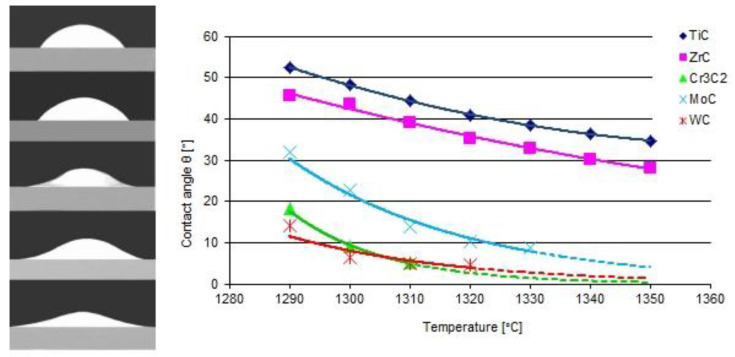
Wettability of carbides with a liquid NiBSi alloy: contours of droplets over flat TiC, ZrC, Cr_3_C_2_, Mo_2_C, and WC substrates at an initial temperature of 1290 °C, and the temperature dependence of the contact angle (average of two trials). The broken line extrapolates the relationship after the completely spread of droplets (θ ≈ 0°).

**Figure 4:**
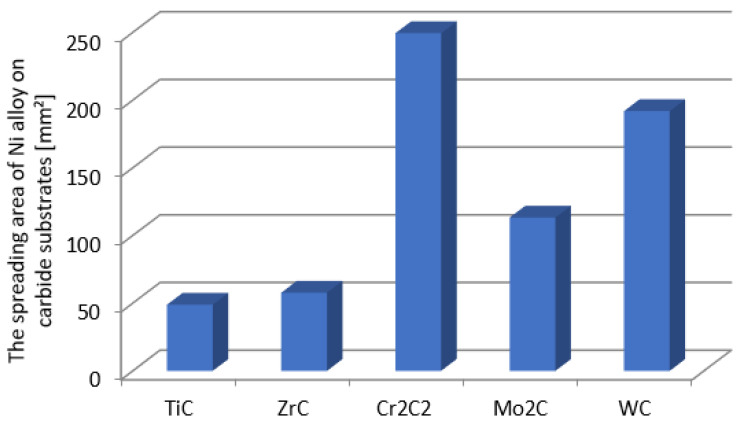
The surface areas of the NiBSi alloy droplets spread over the surface of particular carbides after the end of the entire wettability test (average of two trials).

The results of the model tests are presented in Figure 3 and Figure 4. The first shows the shapes of the liquid NiBSi droplets resting over the carbide substrates captured at 1290 °C, along with the plots of contact angles as a function of the temperature. The results indicate that all of the tested TMCs of the VIB group were more wettable than those of the IVB tranistion metals group. The wettability of the carbides increased (θ decreases) with the temperature increase, which is characteristic for the thermally activated processes [14]. The wettability of ZrC was slightly better than that of TiC. In the VIB group, at the initial 1290 °C temperature, the carbide wettability remained in the following sequence WC > Cr_3_C_2_ > Mo_2_C, but all contact angles droped quickly to a value below 10° with the increase in temperature. However, it should be taken into account that the measurements of small contact angles were subjected to the relatively higher error.

Figure 4 presents the droplet spread surface areas after the whole test. The obtained results were consistent with those of the wettability, and indicated a more intense interaction of the liquid NiBSi alloy with the VIB group TMCs.

### 3.2. Interaction of Carbide Powder Particles with Liquid NiBSi during Surfacing

Studies on the production of SCLs were carried out in a wide range of parameters (Table 3) due to the differences in the physical properties of individual carbides (density, thermal capacity and conductivity, specific heat, etc.). However, only the layers selected for all of the comparative tests described in the article that were obtained at the welding current value shown in Table 4 were considered optimal for the given chemical composition of the padding weld. The criterion was the continuity of the layer along with the uniform distribution of the carbide without metallurgical discontinuities in the form of cracks and porosity in the bulk.

#### 3.2.1. Overlays with Carbides of IVB TM Group

**Figure 5:**
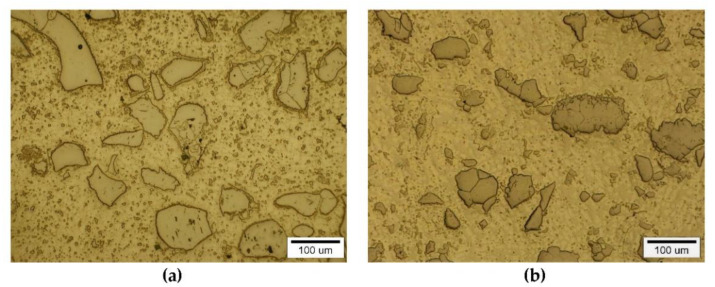
Optical microscope micrograph: Structure of PTAW obtained composite overlays on NiBSi alloy basis reinforced with TiC (**a**) and ZrC (**b**) particles.

**Figure 6:**
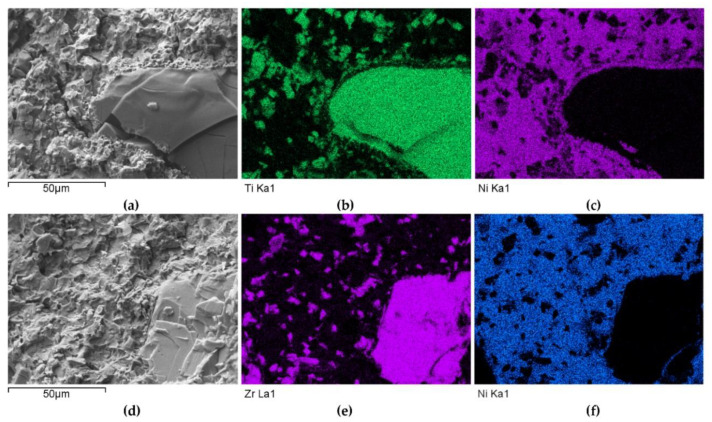
SEM/EDS micrographs of fractured surfaces of NiBSi-TiC (**a**–**c**) and NiBSi-ZrC (**d**–**f**) composite welds enabling phases identification.

The instrumental analysis of the cross sections of the padding welds and their fractured surfaces revealed a composite structure with a specific morphology: diversified shapes and sizes of the reinforced particles were observed. Figure 5a shows large, irregular TiC grains surrounded with a fraction of much smaller particles on the matrix background. A fairly similar grain morphology was visible in the welds containing ZrC (Figure 5b). This was confirmed by the fractographic analysis of both composite layers. The trans-granular cleavage of the reinforcing particles and the relatively ductile inter-granular matrix fracture of the layers are visible in Figure 6.

**Figure 7:**
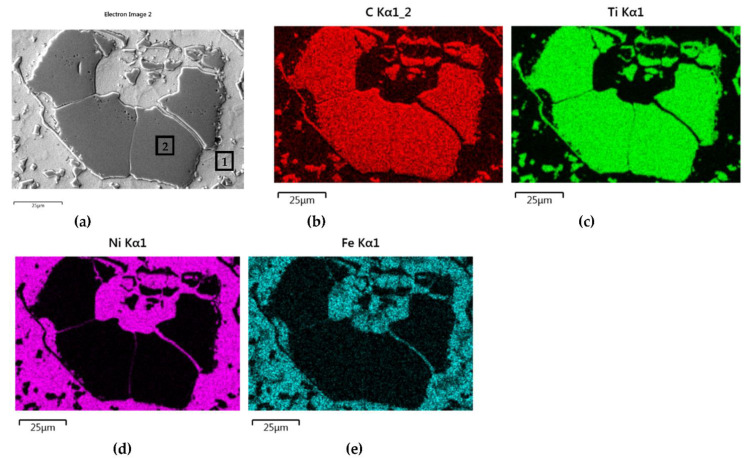
NiBSi-TiC layer: SEM micrograph with points of the quantitative analysis marked (**a**) and surface distributions of C (**b**), Ti (**c**), Ni (**d**), and Fe (**e**).

**Figure 8:**
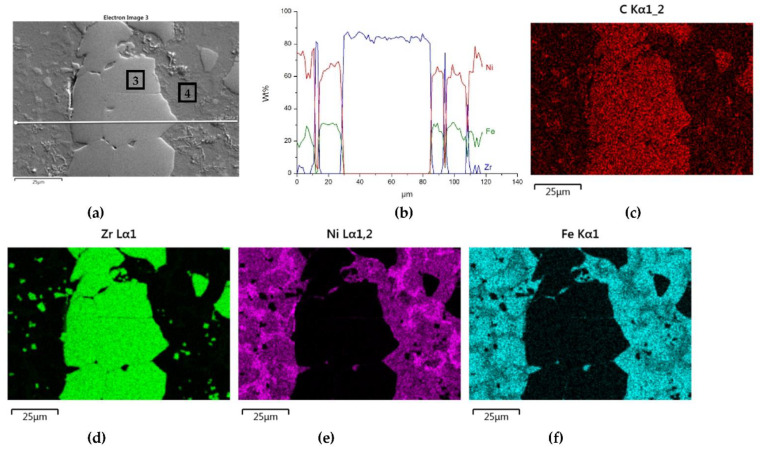
NiBSi-ZrC layer: SEM micrograph with points of the quantitative analysis marked (**a**), linear propagation of components along the marked line (**b**) and EDS maps of C (**c**), Zr (**d**), Ni (**e**), and Fe (**f**) distribution.

**Figure 9:**
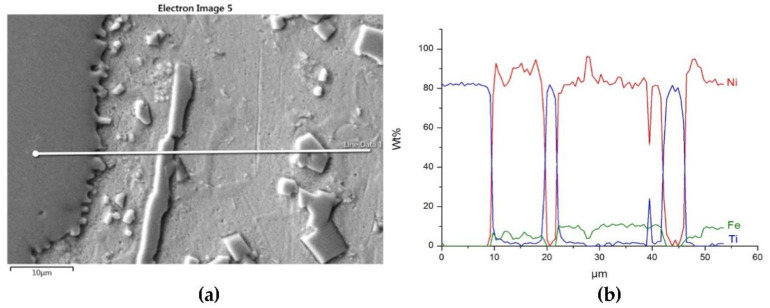
Structure close to the Ni alloy−TiC interfacial boundary (**a**) and concentrations of elements along the marked line (**b**).

The distributions of the main element concentrations comprising the tested system at the layers’ cross-sections are presented in Figure 7 (for TiC) and Figure 8 (for ZrC). The maps for the dissemination of iron, which came from the partially melted steel substrates, are also included. The distribution maps are accompanied by a linear concentration of elements along the lines perpendicular to the interface between the carbide and matrix (Figure 8b and Figure 9).

Sound boundaries without any discontinuities between the matrix and strengthening phase were visible in both cases. No transition layer was visible at the interface of both carbides and the matrix (Figure 7, Figure 8 and Figure 9). The linear distributions of the Ti, Zr, Ni, Fe, and C concentrations along the marked line (shown in Figure 8b and Figure 9b) do not suggest this either, as evidenced by the steep transitions of the curves between the phases.

**Figure 10:**
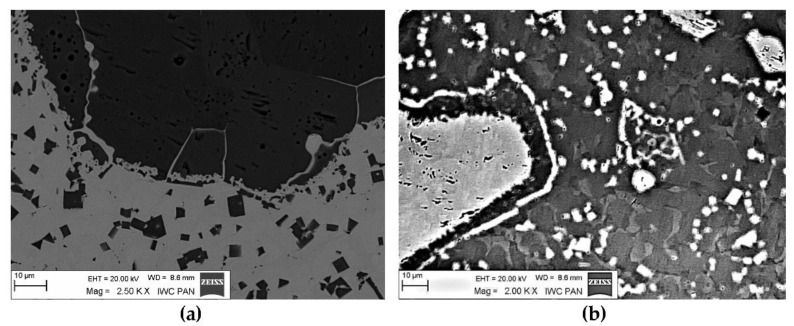
Disintegration of the carbide particle outer layer in the liquid matrix: (**a**) TiC and (**b**) ZrC.

A disintegration of the large TMC (about 80 μm and above) agglomerates was observed, caused by the penetration of the liquid matrix alloy (i.e., Ni-B-Si-Fe) along the grain boundaries during the contact of the solid and liquid phases. This was clearly indicated by photos and the distribution of nickel in Figure 7 and Figure 8. In addition to this breakdown of large TMCs agglomerates into several parts, a splitting of the outer layer of the strengthening phase grains into the much smaller particles is visible in Figure 10. As a result, some amounts of a fine micrometer-order fraction of titanium and zirconium carbides were formed. This surface disintegration was twofold: a narrow band of TiC and ZrC separated from the surface around the large carbide particles, which then broke down into the smaller parts. They dissipated over the entire volume of the liquid through its movement in the welding pool during the process.

The results of the point analysis of elements in the indicated areas (shown in Table 5) reveal that in both cases, except for nickel and silicon (present in the starting powder), a significant quantity of iron existed in the matrix of layers, coming from the partially molten steel substrate. Zr was not present in this area, contrary to a small amount of Ti noticed there. This proved a slight solubility of the last carbide.

#### 3.2.2. Overlays with Carbides of VIB TM Group

**Figure 11:**
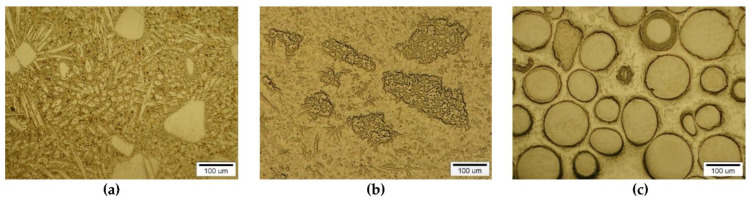
Structure of PTAW obtained composite overlays on NiBSi alloy basis reinforced with Cr_3_C_2_ (**a**), Mo_2_C (**b**) and WC (**c**) particles.

**Figure 12:**
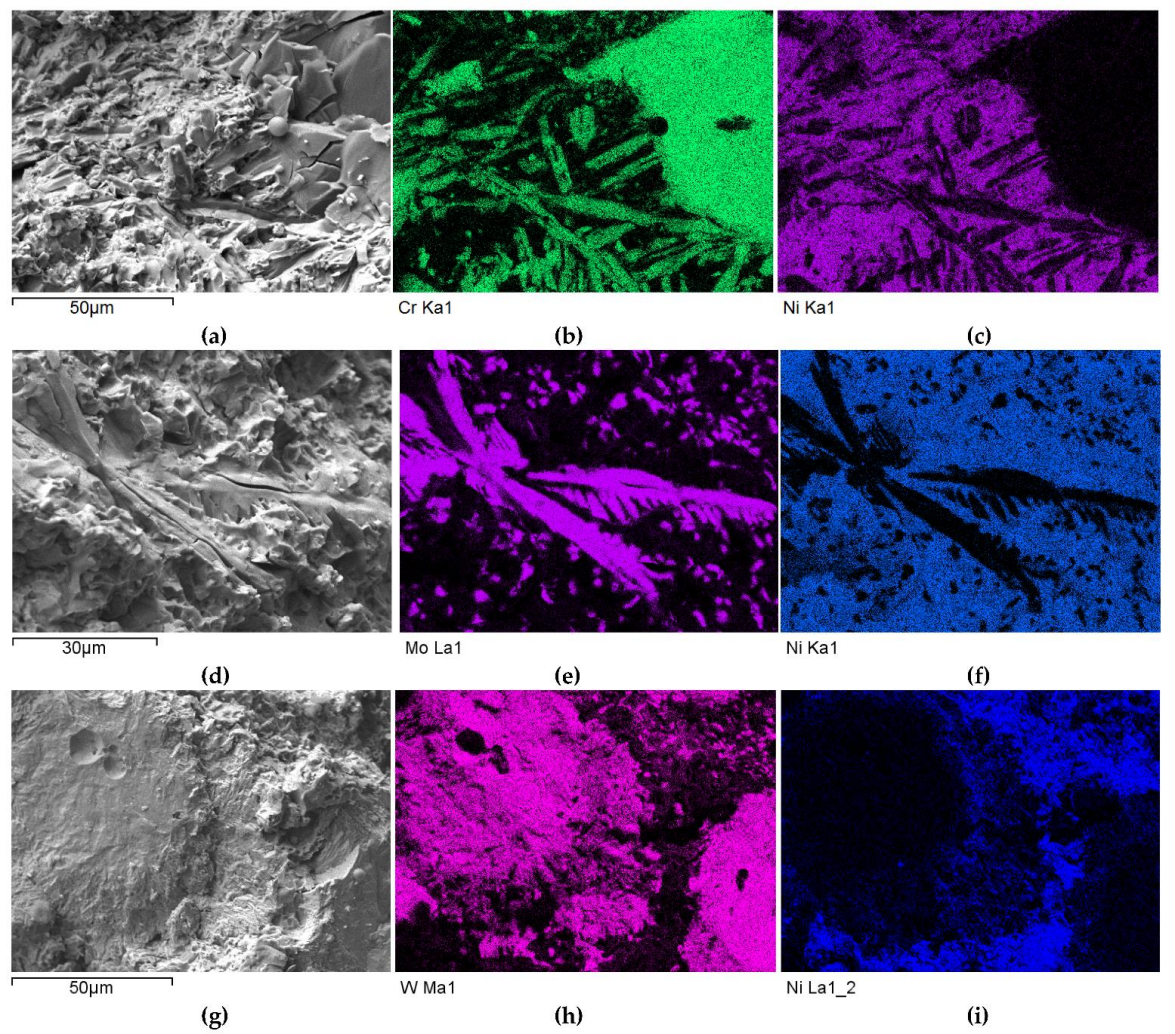
SEM/EDS micrographs of fractured surfaces of NiBSi–Cr_3_C_2_ (**a**–**c**), NiBSi–Mo_2_C (**d**–**f**), and NiBSi–WC (**g**–**i**) composite welds enabling phases identification.

A different nature and intensity of interaction was observed in the SCLs reinforced with TMCs from the VIB group. Beside large irregular grains, much smaller elongated particles of the strengthening phase were visible in the padding welds with Cr_3_C_2_ (Figure 11a and Figure 12a–c). These needle-like particles were presumably Cr_3_C_2_ carbides crystallized from a supersaturated solution. They formed characteristic star-shaped configurations. A little similar morphology was characterized by the molybdenum carbide (Figure 11b and Figure 12d–f). However, the percentage of elongated particles in this case was lower, which may have indicated a reduced solubility of Mo_2_C in the liquid weld pool. Large Mo_2_C particles have clearly developed surfaces. Overlays with WC contain large, nodular carbide particles surrounded by a fine-grained fraction (Figure 11c and Figure 12g–i).

**Figure 13:**
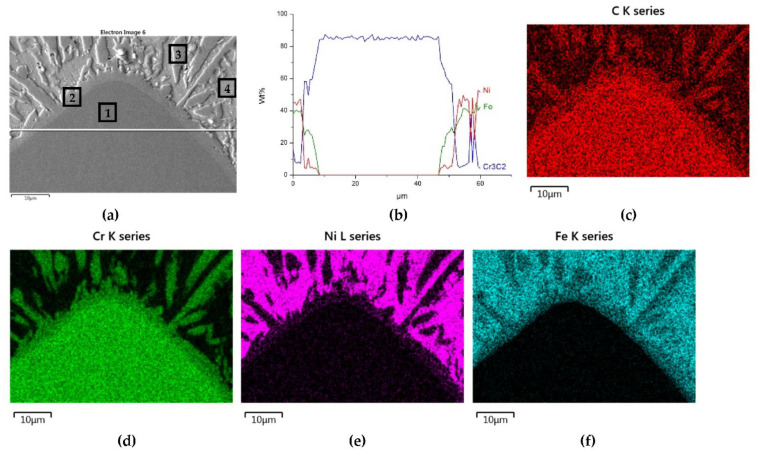
NiBSi-Cr_3_C_2_ layer: SEM micrograph with points of the quantitative analysis marked (**a**), the linear concentration of main components along the marked line (**b**) and the maps of C (**c**), Cr (**d**), Ni (**e**), and Fe (**f**) distribution.

**Figure 14:**
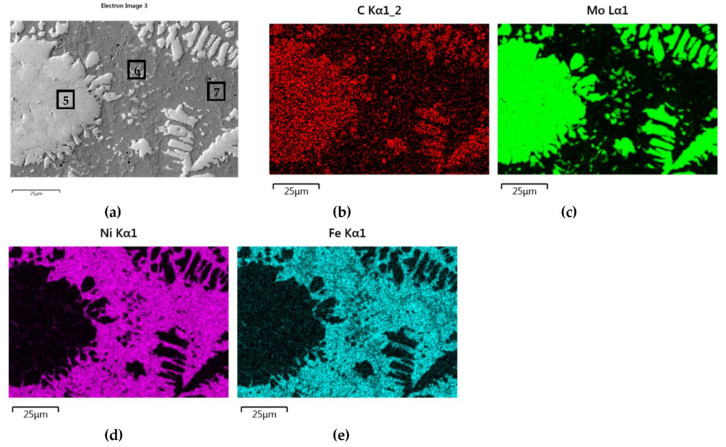
NiBSi–Mo_2_C layer: SEM micrograph with points of the quantitative analysis marked (**a**), and surface distribution of: C (**b**), Mo (**c**), Ni (**d**), and Fe (**e**).

**Figure 15:**
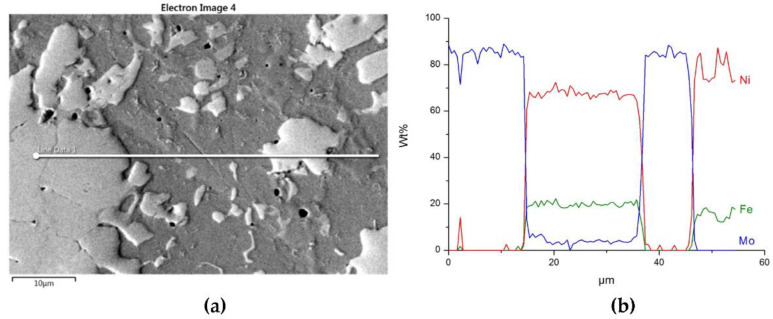
The structure close to the Ni alloy–Mo_2_C interfacial boundary (**a**) and concentrations of elements along the marked line (**b**).

**Figure 16:**
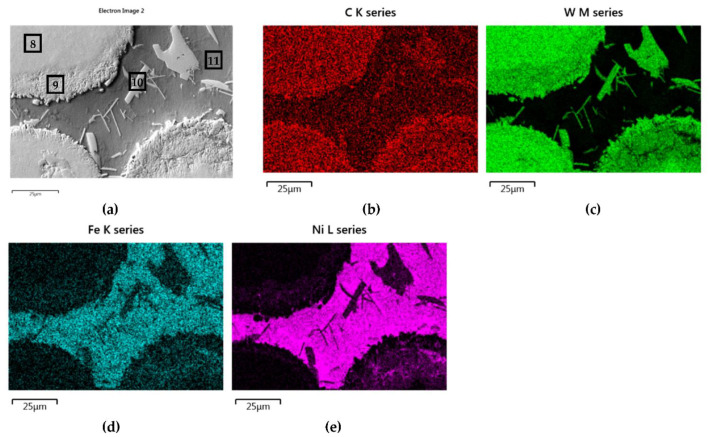
NiBSi–WC layer: SEM micrograph with points of the quantitative analysis marked (**a**), and surface distribution of C (**b**), W (**c**), Ni (**d**), and Fe (**e**).

**Figure 17:**
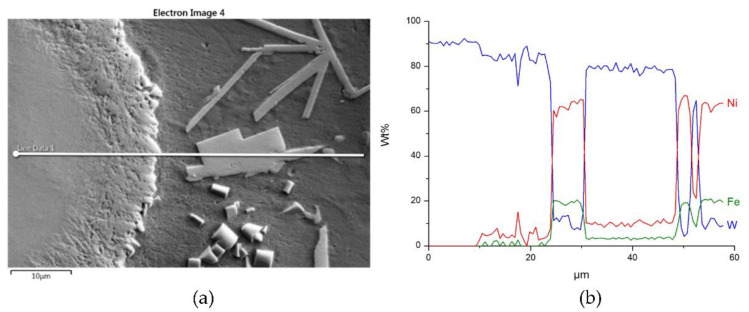
The structure close to the interfacial boundary (**a**) and concentration profiles of Ni, W, and Fe (**b**) in the NiBSi–WC overlay along the marked line.

A set of distribution maps and linear concentrations of components, including the iron allocation, is presented in Figure 13, Figure 14, Figure 15, Figure 16 and Figure 17, the same as for the SCLs with the IVB group TMCs. The results of the point quantitative analysis in selected areas marked in Figure 13a, Figure 14a, and Figure 16a are demonstrated in Table 6.

The mapping, linear elements concentration in the NiBSi–Cr_3_C_2_ coating in Figure 13 and the data from Table 6 exhibit the presence of nickel and iron in the transition zone at the matrix–carbide interface and in the bulk of needle-like precipitates. The matrix is rich in nickel and iron with a small amount of silicon and chromium. The gray shell around the Cr_3_C_2_ particles and precipitates in the matrix are composed of chromium, iron, and nickel with some carbon. The results prove the significant solubility of Cr_3_C_2_ in the liquid ground of the padding weld, from which the needles of the Cr–Fe carbides with the addition of Ni crystallize. The intensity of the phase interaction in the system is also indicated by the formation of wide intermediate zones. The concentration profiles of Ni and Fe suggest relatively long range of diffusion, with Fe being more active.

The structure of Mo_2_C reinforced SCL is shown in Figure 14 and Figure 15. The carbide–matrix interfaces are continuous. The disintegration of grains in the strengthening phase is visible. Precipitations of a new phase against the matrix are detectable. The distribution maps of the elements document the presence of molybdenum in the matrix, which proves the solubility of its carbide during the formation of the coating, confirmed also by the point quantitative analysis (Table 6). However, the Mo fraction in this solution is small.

Figure 16 and Figure 17 show the structure of WC containing SCL and its component distribution. Large, spherical particles of the reinforced phase with continuous interfaces are visible on the matrix background. A wide transition zone is detected around all of them. The maps and linear distributions of the elements’ concentration and the point analysis (Table 6) demonstrate the existence of nickel and iron in both the transition zone and in the weld matrix. The presence of nickel and iron in the transition zone indicates their diffusion into WC. Tungsten is also dissolved in the matrix. Longitudinal secondary precipitations of tungsten carbides with Ni and Fe crystallized out of the matrix solution.

### 3.3. Strengthening Phase Fraction in Overlays

**Figure 18:**
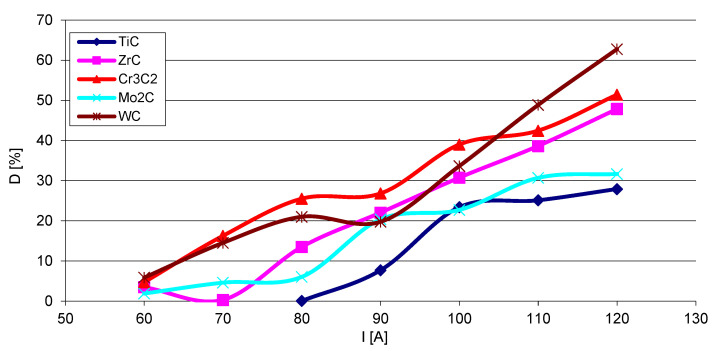
Volume fraction (D) of the substrate material in overlays with particular carbides as a function of the welding current I.

The steel substrate partially melts in the course of surfacing, and the resulting liquid stirs with the welding pool. The fractions (D) of the metal coming from the substrate into SCL were calculated according to Formula (1), where P and S are the cross-sectional areas of the molten substrate and the overlay, respectively. The results show that the fraction of the substrate material in the SCLs significantly increases with the increase of the welding current (Figure 18). The graphs are complex and no clear relationship is visible between D and the position of the given carbide-forming metal in the group of the periodic table of elements. The lowest values of composite matrix dilution with the substrate material are registered for the layers containing titanium and molybdenum carbides.

(1) $$D=\frac{P}{P+S}\;100\%$$

**Figure 19:**
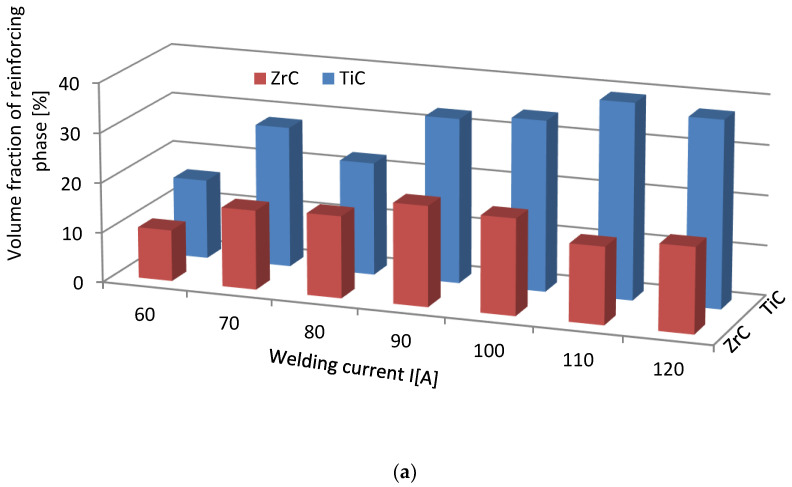

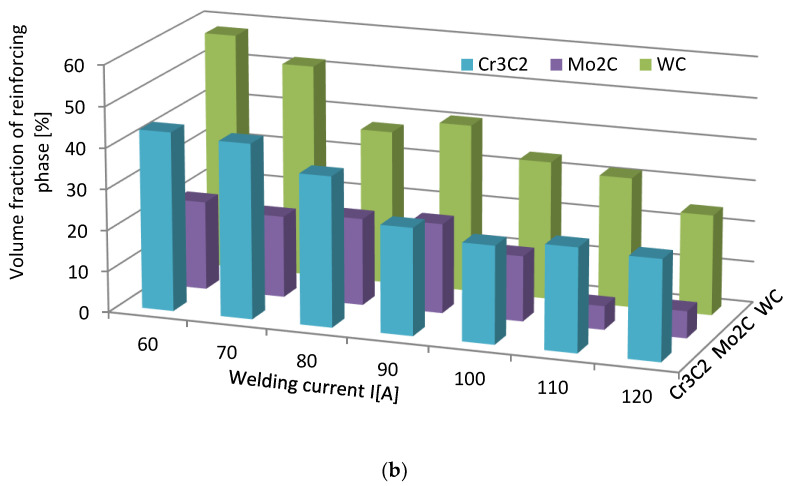
Impact of welding current on the volume fraction of the strengthening phase in overlays with TMs carbides of IVB (**a**) and VIB groups (**b**).

The volume fraction of the strengthening phase in SCLs as a function of the welding current was also analyzed. The measurements were carried out at 100× magnification applying the Cavalieri–Hacquert principle, according to which the ratio of the given phase volumes in the composite is equal to the ratio of the surface areas of these phases at the cross-sections. The obtained results are presented graphically in Figure 19a,b. In the coatings reinforced with TiC and ZrC (group IVB), a systematic increase of fraction with the increasing current intensity was observed, despite the increasing volume of the padding weld due to the increasing fusion of the substrate. The opposite tendency was noted for surfacing welds with carbides of VIB group metals (Cr_3_C_2_, Mo_2_C, and WC). This can be explained by the inferior wettability (less intense interaction with the matrix) group IVB TMCs, which impact their introduction into the liquid. The increase of welding current promotes the thermal activation of the wetting process to maintain larger amounts of TiC and ZrC particles in the liquid. In SCLs reinforced by TMCs of the VIB group, the increase in the linear energy of the welding intensifies the dissolution of carbides, so their fraction decreases with its increase.

### 3.4. Metallurgical Considerations

The results of the experiments show the intense interaction of all TMCs with liquid nickel modified with B and Si additives, both under the stable conditions of the contacting phases and during the dynamic technological process of PTAW, including the melting of powders with a plasma beam, the formation of a liquid solution with the partially molten substrate material, and the crystallization of SCL. As a measure of such an activity, the equilibrium contact angle along with the spread area on a flat surface for the first group of tests was taken, while the formation of SCLs and their structure, with particular emphasis on the vicinity of the interfacial boundary, was taken for the second. The wettability of all of the tested TMCs with the NiBSi alloy was generally good from the initial temperature of 1290 °C, and improved with its increase, as expected. The wettability of group VIB TMCs was better than that of IVB: the contact angles in both groups were close to 0° and in the range 30–40°, respectively (Figure 3). This was sufficient for the technological process. The trend of improved wettability from top to bottom in both TMC groups was observed. The wettability of TMCs had been tested previously, but only for pure Ni [30,31], necessarily at much higher temperatures (T_fNi_ = 1455 °C). The spreadability of the liquid NiBSi alloy over the surface of particular TMCs was consistent with the results of the wettability tests (Figure 4). The results obtained in the study indicate the possibility of producing Ni matrix composites with B and Ni temperature depressants in a much lower temperature range, which is important in massive scale manufacturing.

The structures of the PTAW obtained SCLs were complex and varied for both of TMCs groups studied. The research confirms the different nature of the interaction of the liquid Ni alloy matrix with each TMC. Penetration of the liquid matrix along the grain boundaries of TiC and ZrC took place, resulting in a disintegration of large carbide agglomerates into smaller particles. This is a known mechanism that utilizes the energy surplus of grain boundaries in relation to the interior of the solid phase [14]. If the condition

б_GB_ > б_SL_ cos (ψ/2) (2)

is met (where б_GB_, б_SL_, and ψ are the solid–solid grain boundary energy, solid–liquid interfacial energy, and dihedral contact angle, respectively), the equilibrium in the system was not reached and the liquid flows into the solid along grain boundaries.

The interfacial boundaries in the SCLs of both groups are continuous, with no discrepancies. This means there was compliance with the results of the phase interaction tests in the model studies for small objects with curved surfaces. This also confirms the correctness of the PTAW process carried out, as the contact time of the phases in the liquid pool was sufficient to wet all the surfaces of the solid particles. In the SCLs with the IVB group TMCs there were no transition zones at the boundaries between the carbides and the matrix. TiC is very slightly soluble in liquid, while in the SCLs reinforced with ZrC the presence of Zr in the matrix was not revealed, which proves the complete insolubility of this carbide under the process conditions. In SCLs fortified with VIB TMCs, the Cr_3_C_2_, Mo_2_C, and WC particles partially dissolved and crystallized from the supersaturated solution. In addition, transition zones were formed in these SCLs at the carbide–matrix boundaries, especially in the Cr_3_C_2_- and WC-reinforced coatings. The iron from the molten substrate played an active role in the phases interaction: its presence was noted both in the matrix, including the part separating carbide agglomerates (group IVB), and in transition layers at the interphase boundaries (group VIB). Therefore, during PTAW we are dealing with the active role of three elements: boron, silicon, and iron. A similar mechanism was observed in earlier works [32,33]. The analysis of the double phase equilibrium systems for the combinations of tested TMs with B, Si, and Fe revealed liquid solutions in the entire concentration range and the presence of numerous intermetallic phases for all cases [34]. This shows a tendency towards their mutual interaction.

When comparing the behavior of TMCs during the production of SCLs, one should take into account their electronic structure, physicochemical properties, and the position of the parent TM in the periodic table of elements. Table 7 presents the data relevant from this point of view from many sources. These data are referred to the TMCs of IVB and VIB groups, and also to the not experimentally tested VB group.

TMCs have generally low electrical resistance, decreasing with the group number and from top to bottom inside. This proves the significant participation of TM–TM metallic bonds, in addition to the existing TM–C bonds in these compounds. The structure of TMCs is a metallic lattice structure (fcc for monocarbides, orthorhombic for Cr and Mo, and hexagonal for WC) with the C atoms in the interstitial positions [41]. Therefore, the energy necessary to create the network is reduced due to the inclusion of carbon atoms [44]. TM–C bonds are weakened with the increasing filling of the d-band. This applies to all parent TMs: 3d, 4d, and 5d [37,45]. Thus, the tendency of the metallic character of TMCs increases from left to right and from top to bottom in groups IVB–VIB of the periodic table.

The thermodynamic stability of the TMCs of groups IVB and VB is high, decreasing with the group number from very high for IVB, high for VB, and somewhat softer for the TMCs of group VIB under consideration. The trend of the formation of parent TMs solutions with liquid nickel is similar. This is evidenced by the ΔG_F_ values of the carbide formation and the bond dissociation energies E_BD_, as well as the heat of formation of ΔH_Ni mix_ solutions quoted in Table 7.

**Figure 20:**
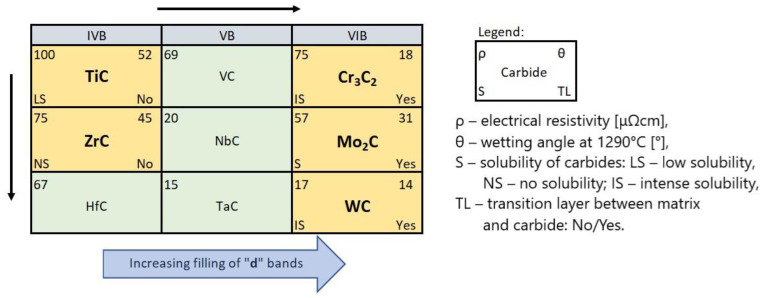
Intensity trends of TMC interactions with the NiBSi matrix during the PTAW formation of SCLs. Each carbide position corresponds to its parent metal location on the periodic table. Carbides subjected to the research are marked.

In summarizing, it can be stated that the interaction of TMCs with the NiBSi matrix during the production of SCLs by the PTAW method tends to increase with the group number of the periodic table, and from the top to bottom inside individual groups. It is presented schematically in Figure 20. The statement is based on the correlations obtained for the two border groups, assuming a specific “interpolation”, and would require verification for the “missing” VB TMCs, which will be the subject of further research.

## 4. Conclusions

Based on the experimental research carried out in the model system and in the real technological process of PTAW, and in the discussion on results, the following can be stated:

1. Carbides of the IVB and VIB TM groups of the periodic table interact with NiBSi alloy, with the increasing intensity related to the group number and the TM location in it. Its measure is the wettability and spreadability, as well as selected aspects of the formation of SCLs: the mechanisms of the disintegration of the strengthening phase in the liquid pool, the solubility of TMCs in the matrix and secondary crystallization, and the formation of transition zones at the interfacial boundaries.
  (a) TMCs of the IVB group are good and these of VIB are perfectly wettable with liquid Ni alloy.
  (b) The fraction of the strengthening phase particles in SCLs and their distribution are related to the interaction of TMCs with the matrix, which is more intense for the VIB group TMCs.
  (c) Particles of TiC and ZrC are decomposed by the penetration of the liquid phase along the grain boundaries. As a result, decomposition of agglomerates on smaller parts occurs and a significant amount of a fine fraction is formed. In contrast with this, Cr_3_C_2_, Mo_2_C, and WC particles are dissolved partially or completely, enriching the matrix in Cr, Mo, and W, respectively. New phases are crystallized from the supersaturated solution when cooling.
  (d) No transition zones are formed at the interfacial boundaries of the IVB group TMCs, unlike in the case of the VIB group TMCs.
  (e) There is a tendency for both of the studied groups of TMCs to intensify the interaction with the Ni alloy matrix, with an increase in the atomic number of the parent metal forming the given carbide.
2. Fe coming from the partially PTAW melted substrate plays an active role in the system, along with temperature depressants B and Si present in the matrix of the composite.
3. The obtained experimental results can be successfully interpreted in light of the electronic structure of TMCs and their physicochemical properties.

## Figures and Tables

**Table 1:** The chemical compositions of NiBSi matrix materials used.

Material		Elements, Wt%				
		C	Si	B	Fe	Ni
Manufacturer’s certificate	Commercial powder	0.03	2.40	1.40	0.40	Bal.
AAS measured	Commercial powder	-	2.18	1.21	0.09	Bal.
	Cast alloy ϕ 3 mm	-	2.45	1.17	0.20	Bal.

**Table 2:** Thicknesses of the deposited carbide coatings (average of five measurements).

Type of Coating	TiC	ZrC	Cr_3_C_2_	Mo_2_C	WC
Coating thickness [μm]	4.7	3.0	2.4	7.8	1.3

**Table 3:** Parameters applied in PTAW surfacing.

Parameters		Value
Main arc current (welding current)		60, 70, 80, 90, 100, 110, and 120 A
Internal arc current		40 A
Plasma arc voltage		25 V
Powder output		6 g/min
Surfacing rate		50 mm/min
Gas flow (argon):	● Plasma generating (orifice) gas	1.5 L/min
	● Shielding gas	8 L/min
	● Powder transporting gas	5 L/min
Oscillation amplitude		8 mm
Oscillating speed		450 mm/min
Plasmatron-welded substrate distance		15 mm
Nozzle diameter		4 mm

**Table 4:** Welding current values used to produce overwelds for comparative testing.

Transition Metals Group	Carbide	Welding Current [A]
IVB	TiC	90
	ZrC	90
VIB	Cr_3_C_2_	90
	Mo_2_C	80
	WC	90

**Table 5:** Point EDS analysis of nickel alloy–TiC and –ZrC regions indicated in Figure 7a and Figure 8a.

Overlay	Region	Elements, Wt%					
		Ti	Zr	C	Ni	Fe	Si
NiBSi–TiC	1	80.8	-	19.2	-	-	-
	2	1.7	-	7.8	79.6	9.2	1.8
NiBSi–ZrC	3	-	82.6	17.4	-	-	-
	4	-	-	4.3	63.8	30.6	1.4

**Table 6:** Results of the EDS quantitative analysis of SCLs with Cr_3_C_2_, Mo_2_C, and WC in points marked in Figure 13a, Figure 14a, and Figure 16a.

Overlay	Region	Elements, Wt%						
		Cr	Mo	W	C	Ni	Fe	Si
NiBSi–Cr_3_C_2_	1	85.3	-	-	14.7	-	-	-
	2	60.2	-	-	10.6	4.3	24.9	
	3	33.1	-	-	9.2	22	35.5	0.3
	4	5	-	-	4.3	50	39.2	1.6
NiBSi–Mo_2_C	5	-	84	-	16	-	-	-
	6	-	4.8	-	7.2	68.7	17.6	1.6
	7	-	1.2	-	7.5	80.7	10.5	
NiBSi–WC	8	-	-	90.6	9.4	-	-	-
	9	-	-	83	10.9	4.5	1.5	-
	10	-	-	79.7	8.2	8.9	3.2	-
	11	-	-	10.3	7.1	61.1	20.2	1.3

**Table 7:** TMCs of the IVB–VIB groups: melting point T_f_ [35], electrical resistivity ρ [35], standard free enthalpy of formation ΔG_F_ [36], formation energy E_TMC_ [37], bond dissociation energy E_BD_ [38], and enthalpy of parent metal solubility in Ni ΔH_Ni mix_ [39].

TM Group	Carbide	T_f_ [°C]	ρ [μΩcm]	ΔG_F_ [kJ/mole C]	E_TMC_ [eV]	E_BD_ [eV]	ΔH_Ni mix_ [kJ/mole]
IVB	TiC	3067 [40]	100	−164.73	−1.62	3.857	−154
	ZrC	3572 [40]	75	−186.11	−1.63	4.892	−236
	HfC	3982 [40]	67	−214.16	−1.88	4.426	−250 [41]
VB	VC_0_._88_	2650	69	−91.40	−0.83	4.109 [42]	−75
	NbC	3610	20	−133.27	−1.06	5.620	n.a.
	TaC	3985	15	−140.70	−1.17	4.975	−133
VIB	Cr_3_C_2_	1810	75	−114.32	n.a.	n.a.	−27
	Mo_2_C	2520	57	−62.13	−0.18 *	n.a.	−32
	WC	2776	17	−34.01	−0.24	4.289 [43]	−14

* value for MoC.

## Data Availability

Experimental methods and results are available from the authors.
